# Occurrence and D-Tryptophan Application for Controlling the Growth of Multidrug-Resistant Non-O157 Shiga Toxin-Producing *Escherichia coli* in Dairy Products

**DOI:** 10.3390/ani12070922

**Published:** 2022-04-04

**Authors:** Mahmoud Elafify, Noha M. Sadoma, Salah F. A. Abd El Aal, Mohamed A. Bayoumi, Tamer Ahmed Ismail

**Affiliations:** 1 Department of Food Hygiene and Control, Faculty of Veterinary Medicine, Mansoura University, Mansoura 35516, Egypt; drmahmoudelafify@mans.edu.eg; 2 Food Control Department, Faculty of Veterinary Medicine, Zagazig University, Zagazig 44519, Egypt; nohasadoma101@gmail.com (N.M.S.); drsalah_aal@yahoo.com (S.F.A.A.E.A.); 3 Department of Clinical Laboratory Sciences, Turabah University College, Taif University, P.O. Box 11099, Taif 21944, Saudi Arabia; t.ismail@tu.edu.sa

**Keywords:** Shiga-toxigenic *Escherichia coli*, antimicrobial resistant, D-amino acid, food stressors, high quality foods

## Abstract

**Simple Summary:**

Milk and dairy products can be contaminated with various fatal foodborne pathogens. Among these pathogens, Shiga toxin-producing *E. coli* (STEC) stains have been incriminated in many foodborne illnesses with severe complications. This study was aimed to investigate the prevalence, serological, and molecular characterization of non O_157_:H_7_ *E. coli* especially Shiga toxin-producing *Escherichia coli* (STEC) in retailed milk and dairy products in Egypt. Furthermore, D-tryptophan was used as natural and safe additive for controlling artificially contaminated dairy products with *E. coli* O_26_:H_11_. The results revealed that 30 out of 150 samples were contaminated with STEC with 64 isolates harbored some virulent genes. Also, these isolates showed a great resistance to various antibiotics. D-tryptophan (D-Trp) at a concentration 40 mM showed a significant (*p* < 0.05) inhibitory effect on *E. coli* O_26_:H_11_ artificially inoculated soft cheese and ice cream. We suggest that D-Trp can be viewed as a promising new line of preservatives for dairy industry.

**Abstract:**

The objectives of the current study were first to determine the prevalence of non O_157_:H_7_ *E. coli*, especially Shiga toxin-producing *Escherichia coli* (STEC) in retailed milk and dairy products in Egypt. Second, the antimicrobial resistance profiling and virulence genes of the isolated *E. coli* strains were screened. Third, evaluation of the inhibitory effects of D-tryptophan against *E. coli* O_26_:H_11_ was further performed. The results revealed that 20% (30/150) of the samples were STEC positive, with 64 isolates harboring some virulent genes, such as *Stx1*, *Stx2*, *eaeA*, and *hylA*. Serological identification revealed four different pathotypes belonging to EPEC, ETEC, EHEC, and EIEC. Antimicrobial susceptibility testing revealed that 100%, 98.44%, 92.19%, 71.87%, 65.63% and 64.06% of the isolates had a resistance against tetracycline, oxacillin, erythromycin, nalidixic acid, sulphamethoxazol, and ampicillin, respectively. D-tryptophan addition (40 mM) to *E. coli* O_26_:H_11_-inoculated soft cheese and ice cream revealed a significant reduction (*p* < 0.05) in bacterial growth, especially when accompanied with other food stressors. D-Tryptophan is considered as an effective food preservative and as a promising alternative candidate in the dairy industry.

## 1. Introduction

One of the most mandatory tasks of food safety sectors, food specialists, and regulatory agencies is food safety, which due to raw food, is incriminated as a severe public health concern when it is contaminated with pathogenic microorganisms [1]. Contaminated foods with foodborne pathogens are incriminated in 300,000 hospitalized cases, with 5000 deaths and nearly USD 7 billion economic losses annually [2,3].

Despite milk and dairy products having a unique composition and a high nutritive value, raw milk and dairy products could provide a potential growth substrate for different zoonotic and foodborne pathogens, which are mostly of animal origin [4]. Hence, the risk of transmission of theses pathogens originating from animal colonization or infection is considerably high [5]. Different organisms of public health concern can potentially contaminate the milk and can have a detrimental effect from a food safety point of view. Among foodborne pathogens, *Escherichia coli* (*E. coli*), especially the toxigenic strains, have gained more attention due to recurrent outbreaks and several incriminations of many foods such as ground beef, raw milk, cheese, and chicken salad [6].

*E. coli* is a Gram-negative, facultative anaerobic bacterium, and is normally an inhabitant of the large intestine of human and animals. The main source of *E. coli* contamination of milk and dairy products is the fecal route during the milking process under poorly hygienic conditions; subsequently, it is considered as a real indicator bacterium for fecal contamination dairy products [7].

In recent years, Shiga toxin-producing *E. coli* (STEC) strains have been incriminated in many foodborne illnesses with fatal complications [2,8]. Their virulence is attributed to their ability to produce cytotoxins named Shiga toxins (*stx1* and *stx2*), in addition to the intimin (*eaeA*) gene that has a vital role in adherence of bacterium with the intestinal epithelium of the host cells, producing clinical symptoms [9]. Several *E. coli* serotypes, besides *E. coli* O_157_:H_7_, were also accused in several outbreaks and produced significant public health hazards, such as *E. coli* O_26_, O_103_, O_111_, and O_145_ [10]. For instance, *E. coli* O_26_ was incriminated in several outbreaks in France in December 2005 [11]. In Australia and Germany, *E. coli* O_26_ serotype was recovered from clinical patients with hemolytic–uremic syndrome (HUS) [12]. Shiga toxin-producing *E. coli* O_103_ was also incriminated in a severe outbreak in Germany due to consumption of contaminated milk during a school trip [13].

In Egypt, raw milk is available to sell directly to consumers, thus contributing to a major health hazard. In addition, several foodborne pathogens have been recovered from this raw milk [14,15,16,17]. Furthermore, some kinds of raw-milk cheeses, such as Kariesh and Damietta soft cheeses, are widely distributed in the Egyptian markets. Kariesh cheese is a home-made raw-milk cheese manufactured via the natural fermentation of milk, especially in rural areas, and it is considered to have main economical outcome and is considered as a source of protein for the farmers and their families. However, it constitutes a significant risk due to the uncontrolled manufacturing method of fermentation [14,16,17]. Damietta soft cheese is another common type of white soft cheese consumed by Egyptian peoples. It is manufactured by focusing on milk coagulation via a coagulating enzyme. Once the curd coagulates, it is cut into small blocks. The curd may be salted first or formed first and then salted [14].

Currently, there is a growing global interest to provide safe, natural, and high-quality processed foods due to the undesirable effects of chemical preservatives and the restriction imposed by food industries on their application. Additionally, thermal treatments have many undesirable effects on the nutritive values of the foods [18,19]. Our previous studies illustrated that D-tryptophan (D-Trp), a type of D-amino acid, has an inhibitory effect on the growth of *L. monocytogenes* under different stress conditions [20]. The antibacterial effect of D-Trp might be attributed to the inhibition of biofilm formation by reducing the initial adhesion between cells and by changing the properties of the extracellular matrix, which has a significant role in protecting the bacteria from environmental insults [21].

To the best of our knowledge, this is the first study that uses D-Trp for reducing the growth of Shiga toxigenic *E. coli* O_26_ in the food models of ice cream and soft cheese, including in the determination of the prevalence, serotypes, antimicrobial resistant profiles, and virulence genes of non O_157_ STEC in milk and some Egyptian dairy products. In addition, a trial for reduction of the viability of *E. coli* O_26_ in treated dairy products with D-Trp was also performed.

## 2. Materials and Methods

### 2.1. Samples Collection

A total of 150 samples including raw milk, ultra-heat-treated (UHT) milk, Kariesh cheese, white soft cheese (*Damietta* cheese), and small-scale ice cream, 30 of each, were collected randomly from various supermarkets and local groceries from December 2020 to July 2021. Raw milk, Kariesh cheese and white soft cheese are widely distributed in the Egyptian markets and have been exposed for selling without any heat treatment. All samples were aseptically collected under hygienic conditions and were kept in an insulated ice box at 4 °C until they reached the laboratory for bacteriological examination on the same day.

### 2.2. Preparation and Isolation of Non O_157_ E. coli

The preparation of samples was carried out according to the protocol described by Roberts et al. [22]. Briefly, 25 g of cheese or warmed ice cream at 40 °C was weighed and homogenized with sterile modified Tryptic Soy Broth (TSB) (Oxoid, CM0989, Hampshire, UK) containing vancomycin at a final concentration of 40 µg/mL, followed by incubation at 37 °C/24 h. For raw milk and UHT milk, the representative samples (25 mL) from each were mixed well with the same broth containing vancomycin at a final concentration of 40 µg/mL and were then incubated at the same temperature.

For sample culturing, a loopful was taken from the enriched sample by a sterile platinum loop and was streaked onto sorbitol MacConkey agar (Oxoid CM0813, Hampshire, UK) supplemented with cefixime and potassium tellurite (Oxoid SR0172E, Wade Road Basingstoke, Hampshire, UK). Then, all plates were incubated at 37 °C/24 h. Colorless colonies with brown centers were picked up as suspected *E. coli* O_157_:H_7_, and the pink colonies were selected as non O_157_ *E. coli*. At least five colonies or more of the available colonies from each plate containing colorless colonies or pink colonies were picked up and purified again on the same medium and then stored in glycerol 15% at −20 °C for further examination and identification.

### 2.3. Serological Identification of the Recovered E. coli Isolates

All recovered *E. coli* isolates were refreshed again onto nutrient agar (Oxoid, Hampshire, UK) and biochemically examined via biochemical tests such as indol, methylred, voges-proskauer, citrate utilization, and hydrogensulphide [23]. All *E. coli* isolates exhibited their characteristics from the used biochemical tests and were serologically identified according to Kok et al. [24] by using rapid diagnostic *E. coli* antisera sets (DENKA SEIKEN Co., Chuo-Ku, Tokyo, Japan) for diagnosis of the Enteropathogenic types. All the procedures were carried out according to the manufacturer’s instructions.

### 2.4. Antimicrobial Susceptibility Testing

The antimicrobial sensitivity phenotypes of the serologically identified strains were examined to identify their sensitivity to the antimicrobial agents using the disk diffusion method [25]. The used antimicrobial agents were cefazolin (CZ) (30 µg); kanamycin (K) (30 µg); imipenem (IPM) (10 µg); cefotaxime (CF) (30 µg); erythromycin (E) (15 µg); ampicillin (AM) (10 µg); ciprofloxacin (CP) (5 µg); nalidixic acid (NA) (30 µg); clindamycin (CL) (10 µg); tetracycline (T) (30 µg); amikacin (AK) (30 µg); oxacillin (OX) (1 µg); gentamicin (G) (10 µg); sulphamethoxazol (SXT) (25 µg). The discs were purchased from Thermo Fisher Scientific (Basingstoke, UK). The sensitivity of the *E. coli* isolates to the antimicrobial discs was determined according to the recommended guidelines established by CLSI [26]. The Multiple Antibiotic Resistance (MAR) index for each isolate was recorded via the following formula described by Singh et al. [27].

MAR index = No. of resistance (isolates categorized as intermediate were considered sensitive)/total number of tested antibiotics.

### 2.5. Molecular Characterization of Non O_157_ E. coli

Genomic DNA extraction from non O_157_ *E. coli* isolates was carried out by the boiling method for 20 min [16,28]. DNA extracts were tested to detect virulent genes: *Stx1*, *Stx2*, *eaeA* and *hylA* at product sizes 614, 779, 890, and 165 bp, respectively. The primer sequences are illustrated in Table 1 [29,30,31]. The amplification was performed using a thermal cycler (Master cycler, Eppendorf, Hamburg, Germany). Amplification conditions consisted of an initial denaturation at 95 °C/3 min followed by 35 cycles of 95 °C for 20 s, 58 °C for 40 s and 72 °C for 90 s, and the final cycle was 72 °C/5 min. The reference strains were *E. coli* O_157_:H_7_ Sakai (positive for *stx1*, *stx2*, *eaeA* and *hylA*) and *E. coli* K12DH5α (negative control strain). The amplified DNA fragments were analyzed by 2% agarose gel electrophoresis (Applichem, Darmstadt, Germany, GmbH) in 1x TBE buffer stained with ethidium bromide and visualized on UV transilluminator.

### 2.6. In Vitro and Food Model Reduction of E. coli O_26_

#### 2.6.1. Preparation of Bacterial Strains

Three Shiga toxins *E. coli* O_26_:H_11_ strains, of food origins, were kindly provided by Prof. Dr. Khalid Ibrahim Sallam, Chairman of the Food Control Department, Mansoura University, Egypt and were used in this experiment. Strains were stored at −80 °C in tryptic soy broth (TSB; Oxoid, Basingstoke, UK) containing 20% glycerol.

For enrichment, a loopful was taken from each strain and was individually inoculated into a sterile TSB followed by incubation for 24 h at 37 °C. Then, a loopful was streaked onto tryptic soy agar (TSA; Oxoid, Basingstoke, UK) and incubated at 37 °C for 24 h. A single purified colony from each strain was selected individually and inoculated into a sterile TSB and incubated at 37 °C/18–24 h to obtain a final concentration of approximately 10^9^ CFU/mL. Then, an equal volume from each strain (1 mL) was gathered and combined in a sterile plastic tube (10 mL) and mixed well using a vortex. This mentioned method is called the cocktail pathogens protocol [20].

#### 2.6.2. Reduction of *E. coli* O_26_ in Broth Media Supplemented with D-Trp at Chilled Stress (4 °C) and at Room Temperature (25 °C)

Initially, for determination of the inhibitory concentration of D-Trp, a basal broth medium containing peptone, yeast and glucose (PYG) was used. Approximately, 100 µL aliquot from the diluted bacterial culture (10^7^ CFU/mL) was inoculated into 900 µL of sterile broth previously inoculated with D-Trp (Sigma-Aldrich, Gillingham, CO, USA) at concentrations of 20, 30 and 40 mM and was stored at 4 °C for 4 weeks. At each week, 100 µL of the treated and control groups was cultured onto sterile TSA and incubated at 37 °C/18–24 h, and the survival bacterial counts were determined using a colony counter. Three independent trials, with three samples for each time point, were performed at each concentration of D-Trp.

According to the preliminary results obtained from the antimicrobial activity of D-Trp at the chilled stress (4 °C), we used the significant inhibitory concentration (40 mM) of D-Trp and inoculated it into PYG media at 25 °C/4 days without any stress condition to investigate if the D-Trp has an inhibitory effect against *E. coli* growth or not. Each day, the survival bacterial counts were determined by the same mentioned manner. Three independent trials with three samples for each time point were performed.

#### 2.6.3. Preparation of Dairy Products and Inoculation with Shiga toxin *E. coli* O_26_ and D-Trp

Damietta soft cheese was prepared according to Youssef et al. [32]. Briefly, cow’s raw milk (3% fat, *w*/*w*) was collected from a dairy farm located in Mansoura City, Egypt, which was heat treated at 65 °C/30 min in a water bath containing recycled water. Then, when the milk was cooled at 40 °C, calcium chloride (0.02 g/L), sodium chloride (1.5% and 3%), and a cocktail of pathogens of *E. coli* O_26_:H_11_ at a concentration of 10^6^ CFU/mL (10^9^ CFU/mL was diluted into 1 L of milk to obtain 10^6^ CFU/mL) was added to the warmed milk and was distributed into sterile vats. Then, the rennet (0.2 g/L) and D-Trp (40 mM) were added and incubated at 37 °C/45–60 min. Control cheese was manufactured as described above but without D-Trp. Once the curd was obtained, the curd was separated from the whey and stored at 4 °C in clean sterile containers for bacteriological analysis. The used concentration of D-Trp was selected according to the significant results from the previously inoculated broth.

Vanilla ice cream powder (80 gm) was purchased from a local supermarket in Mansoura City, Egypt. Its ingredients were sugar, milk protein, emulsifier (lactic acid ester E472), vegetable fats (palm kern oil–coconut oil), stabilizer (Guar gum E412) and vanilla. Ice cream manufacturing was performed according to the label’s instructions. Briefly, the content of the ice cream bag (80 gm) was placed onto a sterile plastic container, and 200 mL of UHT (Ultra-heat treated) milk as well as the predetermined concentration of D-Trp was poured onto it with, was mixed well, and the diluted cocktail pathogens were added to obtain a final concentration 10^5^ CFU/mL. Then, the ice cream was distributed into small sterile plastic containers and stored at −20 °C. The same procedure was followed for the control ice cream but without D-Trp.

To determine the viable counts of *E. coli* O_26_:H_11_. Each week, twenty-five grams of the melted ice cream or cheese was mixed with 225 mL of sterile BPW (Buffer–peptone–water), and then a ten-fold serial dilution was performed using 0.1% peptone water, and the surface spreading method was performed. All the recovered colonies were counted, and the results were expressed as log_10_ CFU/gm. Three independent trials, with three samples for each time point, were performed.

### 2.7. Statistical Analysis

The recovered data were transformed into log CFU/mL or CFU/g. The data of the triplicate samples were averaged. One-way ANOVA tests were used to determine the significant difference between the control samples and treated samples with different concentrations of D-Trp. Furthermore, a *t* test (*p* < 0.05) was used to investigate the significant difference between treated samples at the determined inhibitory concentration and the control one. The statistical analyses were carried out using the computer statistic program IBM SPSS Statistics for Windows, Version 19.

## 3. Results

### 3.1. Prevalence and Serological Identification of the Recovered Isolates

The presented data in our study illustrated that 9, 11, 2, and 8 of the market raw milk, Kariesh cheese, white soft cheese, and small-scale ice cream samples were contaminated with *E. coli*, respectively (Table 2).

Ten different serotypes were recovered from the examined samples. The most predominant pathotypes were enterohaemorrhagic *E. coli* (27/64; 42.19%), followed by enteropathogenic *E. coli* (23/64; 35.94%), enterotoxigenic *E. coli* (8/64; 12.5%), and finally enteroinvasive *E. coli* (6/64; 9.37%) (Table 3).

### 3.2. Antimicrobial Resistance and Molecular Characterization of E. coli

**Figure 1:**
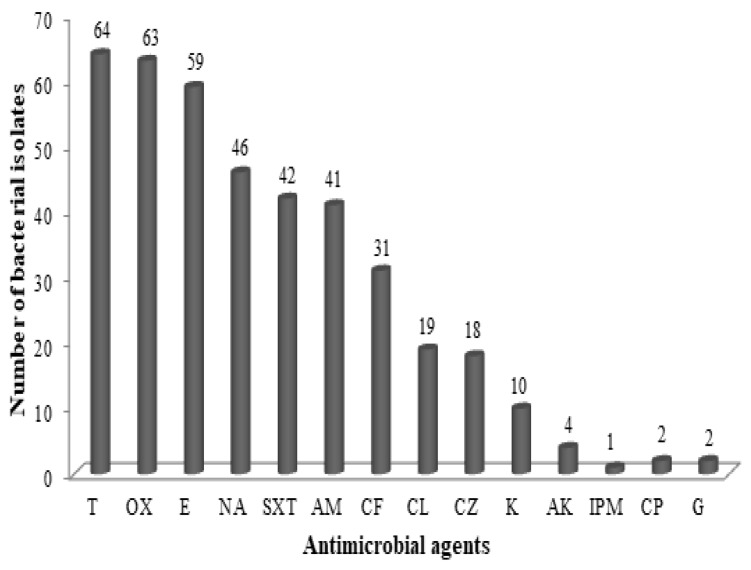
Antibiotics resistance of *Escherichia coli* isolated from milk and dairy products. T: Tetracycline; OX: Oxacillin; E: Erythromycin; NA: Nalidixic acid; SXT: Sulphamethoxazol; AM: Ampicillin; CF: Cefotaxime; CL: Clindamycin; CZ: Cefazolin; K: Kanamycin; AK: Amikacin; IMP: Imipenem; CP: Ciprofloxacin; G: Gentamicin.

All recovered isolates were screened for their resistance to antimicrobial agents. The obtained results revealed that 100% of the isolates were resistant to tetracycline. Likewise, above 65% of the recovered isolates had multidrug resistance to several antimicrobial agents such as oxacillin (98.44%), erythromycin (92.19%), nalidixic acid (71.87%), and sulphamethoxazol (65.63%). Less than 50% of the isolates revealed a resistance to other antimicrobial agents as shown in Figure 1.

Regarding the virulent genes of the recovered isolates, all recovered isolates were molecularly examined for determining their virulence via detection of some virulent genes, such as *Stx1*, *Stx2*, *eaeA*, and *hlyA* (Table 1 and Table 4). The present data showed that 58, 47, 21 and 24 isolates harbored the respectively mentioned genes. Furthermore, our results revealed that all *E. coli* O_26_:H_11_ and O_111_:H_2_ isolates expressed the four examined virulent genes (Table 4).

### 3.3. The Inhibitory Effects of Different Concentrations of D-Trp against E. coli O_26_:H_11_ Inoculated into PYG Broth at 4 °C and 25 °C

**Figure 2:**
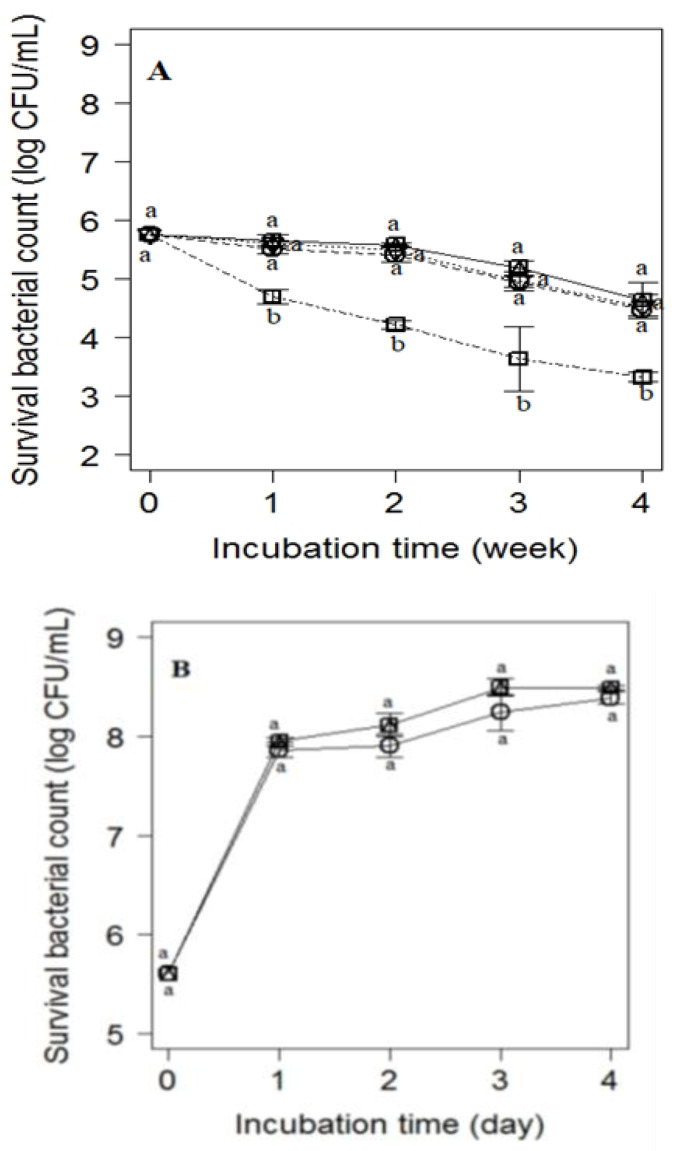
Inhibitory effect of D-tryptophan on the viable counts of *E. coli* O_26_ inoculated into PYG media (**A**) at 4 °C at various concentrations of D-Trp. 0 mM (solid line), 20 mM (dotted line), 30 mM (dashed line), and 40 mM (dot-dashed line), (**B**) at room temperature (25 °C) at 40 mM D-Trp (0 mM D-Trp, □; 40 mM D-Trp, Ο). Values are the mean ± standard deviation of three independent trials. Values with different letters are significantly different (*p* < 0.05).

The effects of D-Trp on the growth activity of the inoculated *E. coli* O_26_ is demonstrated in Figure 2. The results showed that the concentrations of D-Trp < 40 mM did not have any significant inhibitory effect on *E. coli* O_26_ growth. D-Trp at a concentration of 40 mM exhibited a significant reduction (*p* < 0.05) by 1.5 log CFU/mL for *E. coli* O_26_ growth during the four-week incubation period compared with the control broth.

Regarding the inhibitory effect of D-Trp (40 mM) on *E. coli* growth at 25 °C, our results revealed that D-Trp at a concentration of 40 mM did not exhibit a significant reduction (*p* < 0.05) on *E. coli* growth during the incubation period at 25 °C compared with the control broth. This indicates that the stress condition is a vital factor for elicitation of the antimicrobial activity of D-Trp (Figure 2).

### 3.4. The Effect of D-Tryptophan at 40 mM on Experimentally Inoculated Shiga Toxigenic E. coli O_26_:H_11_ Strains on Soft Cheese and Ice Cream under Osmotic and Freezing Stressors

**Figure 3:**
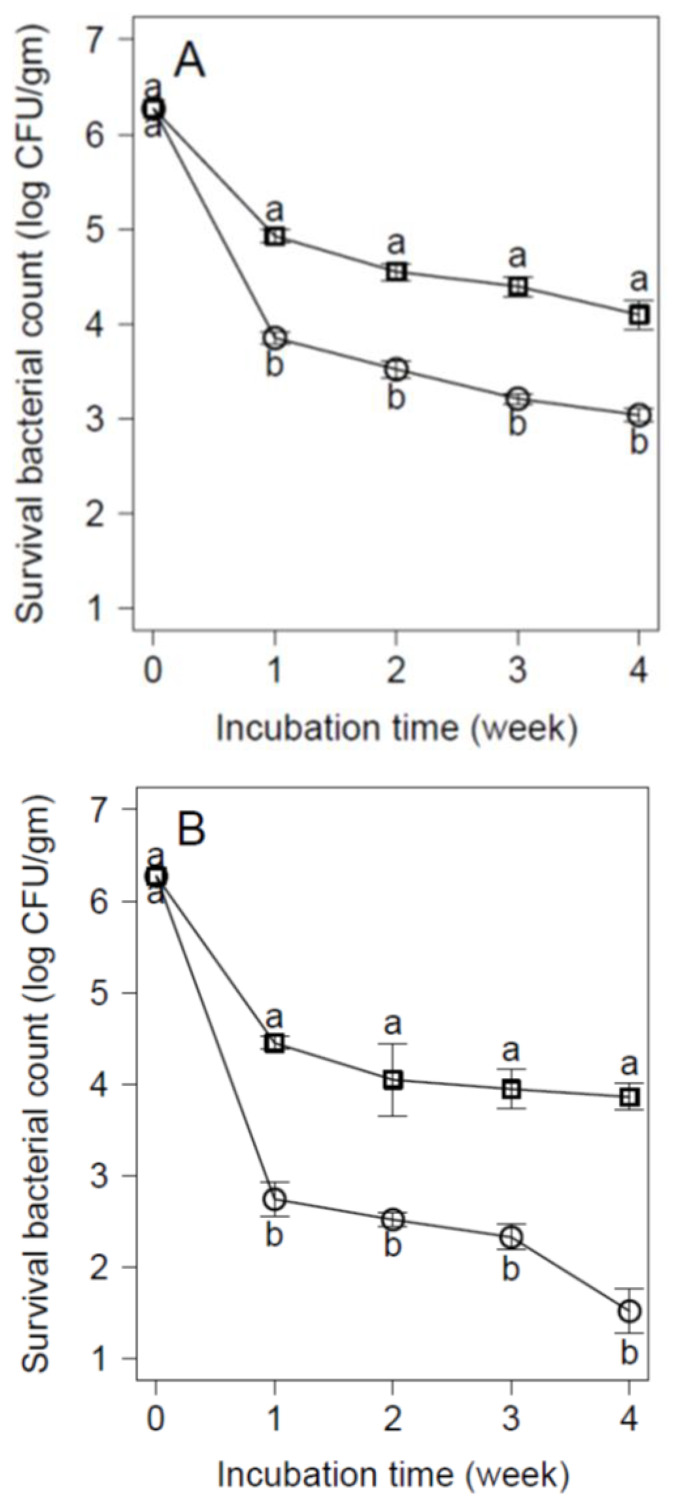
Inhibitory effect of D-tryptophan (0 mM D-Trp, □; 40 mM D-Trp, Ο) on the viable counts of *E. coli* O_26_ inoculated into cheese with (**A**) 1.5% NaCl and (**B**) 3% NaCl at 4 °C. Values are the mean ± standard deviation of three independent trials. Values with different letters are significantly different (*p* < 0.05).

The inhibitory effect of D-Trp at 40 mM was evaluated on an artificially contaminated soft cheese containing 1.5% and 3% NaCl and stored at 4 °C as well as in ice cream stored at −20 °C. The obtained results revealed that D-Trp revealed a lower reduction in *E. coli* viability in contaminated cheese containing 1.5% NaCl by 1 log CFU/g during a 4-week incubation period at 4 °C. Meanwhile, increasing the salt content to 3% NaCl to create osmotic stress in the contaminated cheese with an addition of 40 mM D-Trp caused a more significant (*p* < 0.05) suppression of *E. coli* growth by 1.5 log CFU/g within a two-week incubation period compared with the control samples. These reductions were continued until they reached approximately 2.5 log CFU/g within the four-week incubation period at 4 °C (Figure 3).

**Figure 4:**
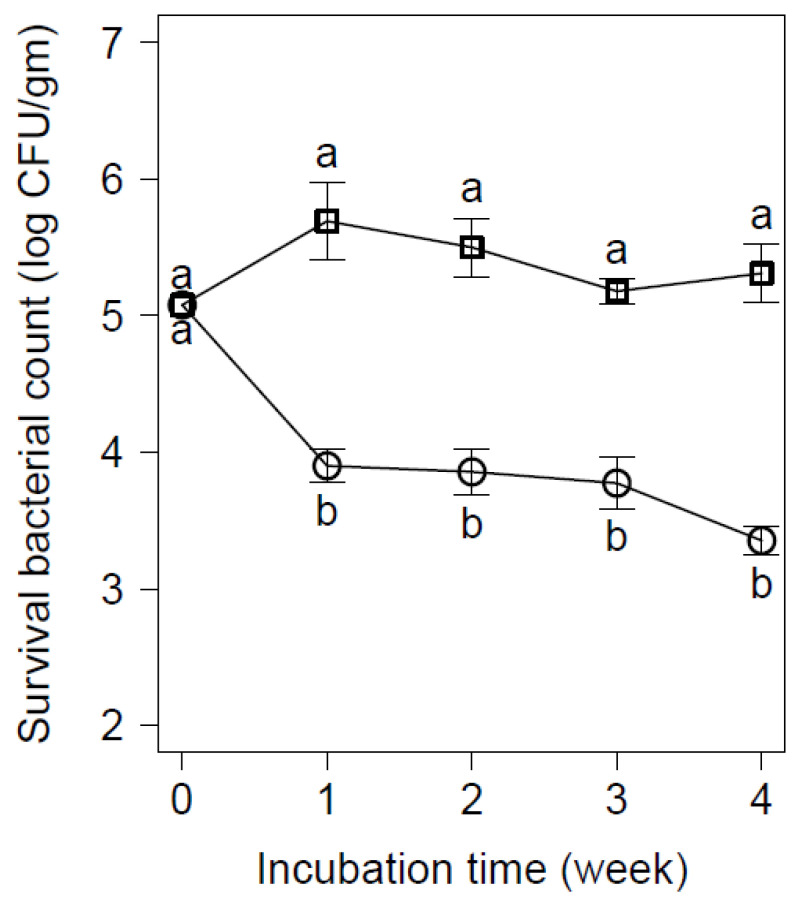
Inhibitory effect of D-tryptophan (0 mM D-Trp, □; 40 mM D-Trp, Ο) on the viable counts of *E. coli* O_26_ inoculated into vanilla ice cream at −20 °C. Values are the mean ± standard deviation of three independent trials. Values with different letters are significantly different (*p* < 0.05).

Regarding the ice cream, the results showed a significant (*p* < 0.05) reduction of *E. coli* counts, within the two- and four-week incubation period, by 1.7 and 2 logs CFU/g, respectively (Figure 4).

## 4. Discussion

One of the most important strategies to extend the shelf life of foods and to avoid public health hazards is the application of natural and safe antimicrobial additives. Some D-amino acids have a therapeutic potential effect and antimicrobial activity. For instance, D-serine was identified as a neurotransmitter agent besides its antimicrobial activity under osmotic stress [33]. To the best of our knowledge, this is the first study that used D-Trp as a natural D-amino acid for controlling the growth of *E. coli* O_26_:H_11_ in dairy. The achieved results regarding the prevalence of *E. coli* in dairy indicated that market raw milk, Kariesh cheese, white soft cheese and small-scale ice cream were contaminated with STEC. The results of the present study are in agreement with a study conducted by Elafify et al. [2] who recorded that 10 samples of raw milk were contaminated with *E. coli*. Ombarak et al. [34] stated that 14 milk samples were contaminated with *E. coli*. However, some studies have reported a higher prevalence than our study. Hassan et al. [35] reported that 75% of the examined milk samples were contaminated with *E. coli* in Beni-suef, Egypt.

In Egypt, reports highlight the importance of raw-milk Kariesh cheese as a popular food matrix that contributes to a potential health hazard for consumers [16,36]. The obtained results illustrated that out of 64 *E. coli* isolates, 25 isolates (39.06%) recovered from Kariesh cheese were positive for STEC. Similarly, 42.7% of *E. coli* isolates recovered from Kariesh cheese in Egypt were positive for STEC [37].

Unsurprisingly, market UHT milk was free from any contamination, suggesting that efficient hygienic, sanitary quality, and proper heat treatment of milk was adopted as previously reported elsewhere [38].

Regarding the ice cream, eight samples revealed contamination with *E. coli*. Several studies also recorded *E. coli* contamination of ice cream with various rates ([2] at 0%; [39] at 2% [40] at 92%). This difference in the isolation rates might be attributed to different procedures used for the packaging, manufacturing, and marketing of ice cream [41].

The abuse and random usage of antimicrobials during animal farming and livestock production might lead to the development of antimicrobial resistance [16]. Our results illustrated that above 50% of the isolates had antimicrobial resistant. For instance, 100%, 98.44%, 92.19%, 71.87%, 65.63% and 64.06% had a resistance against tetracycline, oxacillin, erythromycin, nalidixic acid, sulphamethoxazol, and ampicillin, respectively. These results are in agreement with Kasem et al. [42], who recoded multidrug resistance of *E. coli* isolates recovered from Egyptian dairy products to nalidixic acid (94.1%) and sulphamethoxazol (47.1%). El Bagoury et al. [43] reported that 100% of *E. coli* strains isolated from cheese had a resistance to erythromycin.

On the contrary, Rahimi et al. [44] recorded a lower resistance of *E. coli* strains recovered from the cheese sold in Iranian markets to erythromycin (33.3%), nalidixic acid (1.1%), and tetracycline (11.1%). Furthermore, Gundogan and Avci [45] reported that in Turkey, most *E. coli* isolates recovered from dairy products had a resistance to ampicillin (90.5%) and penicillin (82.1%). These differences among MDR *E. coli* might be associated with dissimilarities in the antimicrobials used at the regional level [42].

The pathogenicity of *E. coli* mainly attributed to some virulence factors such as Shiga toxins, adhesion protein intimin (*eae*), and enterohemolysin (*Ehly*). All recovered isolates were molecularly examined for determining their virulence via detection of some virulent genes such as *Stx1*, *Stx2*, *eaeA*, and *hlyA*. The present data showed that 58, 47, 21 and 24 isolates harbored the mentioned genes, respectively.

Several studies have reported *Stx* genes in milk and dairy products, similar to Elafify et al. [2], who recorded that 30.55% of raw milk was positive for *Stx* genes. Furthermore, Elhadidy and Mohammed [28], Fach et al. [46], and Zweifel et al. [47] recorded that 14%, 30.5%, and 5.7% of raw-milk cheese was positive for *Stx* genes, respectively. Additionally, the *Stx* genes were detected in dairy products with various rates in different countries such as 13% in France [48], 6% in Brazil [49], and 3.9% in Germany [50].

Furthermore, 32.8% and 37.5% of our isolates harbored *eaeA* and *hlyA* genes, respectively. Similarly, the *eaeA* gene was recovered from dairy products at 36% [51]. Various degrees of *hlyA* and *eaeA* genes were recorded in other studies, such that 0.9% and 9.1% of the examined raw milk was positive for the *eaeA* gene in Egypt and Saudi Arabia, respectively [37,52]. In general, the differences in prevalence rates of the *E. coli* strains and their virulence genes might be attributed to sampling density, isolation procedure, and country [53].

In the present study, we evaluated the potential impact of D-Trp, as a new natural food preservative combined with different stress conditions, on the growth of *E. coli* O_26_:H_11_ in different inoculated dairy products to overcome the toxic effect of chemical preservatives and to minimize the negative impacts of the thermal treatment on the nutritive value of the foods. D-Trp at the used concentration did not show any deviations in the sensory characteristics of the soft cheese or ice cream (data not shown). The effect of D-Trp on numerous Gram-negative bacteria in broth has been studied [54]. However, currently, no data are available regarding the effect of D-Trp on *E. coli* in some Egyptian dairy products. *E. coli* O_26_:H_11_ strains were selected for our experiment due to several scientific reasons: *E. coli* O_26_:H_11_ has been incriminated in several outbreaks; clinical patients with HUS have recovered from it; the pathogenicity of *E. coli* O_26_ is not less than *E. coli* O_157_ regarding HUS and renal failure; last but not least, *E. coli* O_26_ has revealed more resistance to food stressors such as acid and salt versus *E. coli* O_157_ [11,12,28].

The effect of D-Trp on the growth activity of *E. coli* O_26_ in broth is demonstrated in Figure 2. The results show that the concentrations of D-Trp < 40 mM were not revealed to have a significant (*p* < 0.05) inhibitory effect on *E. coli* O_26_ growth in the contaminated broth. D-Trp at a concentration of 40 mM exhibited a significant reduction (*p* < 0.05) by 1.5 log CFU/mL for *E. coli* O_26_ growth during the four-week incubation period compared with the control broth at 4 °C.

Regarding the inhibitory effect of D-Trp (40 mM) on *E. coli* growth at 25 °C, our results revealed that D-Trp at a concentration of 40 mM did not exhibit a significant reduction (*p* < 0.05) on *E. coli* growth during the incubation period at 25 °C compared with the control broth. These findings agree with those reported recently [20,54]. The researchers found that D-Trp at 40 mM had a noticeable effect on pathogen growth under food-related stressors.

According to the preliminary data about the inhibitory effect of D-Trp at 40 mM on *E. coli* O_26_ contaminated broth, D-Trp at a concentration of 40 mM was used as an antimicrobial D-amino acid to suppress the growth of *E. coli* O_26_ in artificially contaminated cheese and ice cream. Our findings revealed that D-Trp revealed a reduction in *E. coli* viability in contaminated cheese containing 1.5% and 3% NaCl by 1 and 2.5 log CFU/g during a 4-week incubation period at 4 °C, respectively. A similar finding was reported by Chen et al. [55], who found that increasing concentrations of NaCl (3–5%) with 40 mM D-Trp elicited bactericidal effects on *Vibrio* spp. growth in oysters.

The synergistic effect of D-Trp with freezing stress in inoculated vanilla ice cream has provoked a significant (*p* < 0.05) reduction in *E. coli* O_26_:H_11_ by 2 CFU/g during the four weeks of treatment. Such a reduction in the bacterial population could be attributed to the freezing stress, which enhances the inhibitory effect of D-Trp in ice cream. This finding was similar to that given by Chen et al. [55], but the authors used D-Trp combined with osmotic stress to control the growth of *Vibrio* spp. in oysters, while Elafify et al. [20] tested the effect of D-Trp combined with chilled stress to inhibit *L. monocytogenes* growth in milk. Other reports have tested D-amino acids to control the growth of *S. aureus* via inhibition of biofilm formation [56]. Meanwhile, Rumbo et al. [57] have assessed the antimicrobial activity of several D-amino acids against *Acinetobacter baumannii* and *Pseudomonas aeruginosa* and found that D-amino acids could have inhibitory effects on bacterial growth as well as potential roles in inhibition of biofilm formation.

## 5. Conclusions

The obtained results of the present study revealed the isolation of multidrug-resistant STEC from milk and dairy products retailed in Egypt that contribute to life-threatening situations for consumers. Furthermore, the obtained data revealed a lack of hygienic measures adopted during the milking, processing, and distribution of such dairies. In addition, D-Trp could be a promising tool for dairy preservation and for controlling the growth of the tested Gram-negative foodborne pathogens under stressful conditions. The inhibitory effect of D-Trp with dairy-related stressors might be a safety barrier to minimize the growth of pathogens in food and to avoid the related public health hazards.

## Figures and Tables

**Table 1:** Oligonucleotide primers adopted for amplification of the various virulent genes in *E. coli*.

Primers	Oligonucleotide Sequences (5′ → 3′)	Product Sizes (bp)	References
*stx1* (F)	5′ ACACTGGATGATCTCAGTGG 3′	614	[29]
*stx1* (R)	5′ CTGAATCCCCCTCCATTATG 3′		
*stx2* (F)	5′ CCATGACAACGGACAGCAGTT 3′	779	
*stx2* (R)	5′ CCTGTCAACTGAGCAGCACTTTG 3′		
*eaeA* (F)	5′ GTGGCGAATACTGGCGAGACT 3′	890	[30]
*eaeA* (R)	5′ CCCCATTCTTTTTCACCGTCG 3′		
*hylA* (F)	5′ ACGATGTGGTTTATTCTGGA 3′	165	[31]
*hylA* (R)	5′ CTTCACGTGACCATACATAT 3′		

**Table 2:** Prevalence of *E. coli* in milk and dairy products.

Type of Dairy Samples	Number of Samples	Positive Samples	
		No.	%
Market raw Milk	30	9	30
UHT milk	30	0	0
Kariesh cheese	30	11	36.67
White soft cheese	30	2	6.67
Small-scale ice cream	30	8	26.67
Total	150	30	20

**Table 3:** Serological identification of *E. coli* isolated from milk and some dairy products.

Pathotypes	Serotypes	No. of Strains	Distribution of *E. coli* Isolates				
			Market Raw Milk	Ultra-Heat Treated Milk	Kariesh Cheese	White Soft Cheese	Small-Scale Ice Cream
EPEC	O17:H18	4	-	-	1	-	3
	O114:H4	2	-	-	2	-	-
	O119:H6	13	3	-	4	1	5
	O121:H7	3	-	-	3	-	-
	O146:H21	1	-	-	-	1	-
ETEC	O128:H2	8	1	-	2	1	4
	O26:H11	15	3	-	8	3	1
EHEC	O55:H7	6	1	-	-	2	3
	O111:H2	6	2	-	2	2	-
EIEC	O159	6	3	--	3	-	-

EPEC: Enteropathogenic *E. coli*; ETEC: Enterotoxigenic *E. coli*; EHEC: Enterohaemorrhagic *E. coli*; EIEC: Enteroinvasive *E. coli.*

**Table 4:** Antimicrobial resistance profile and virulent genes of *E. coli* strains (*n* = 64).

No.	Isolates ID	*E. coli* Strains	Type of Dairy Products	Antimicrobial Resistance Profile	MAR Index	Virulent Fenes			
						*Stx1*	*Stx2*	*eaeA*	*hlyA*
1	5	O_26_:H_11_	Market raw milk	T, OX, E, NA, SXT, AM, CF, CL, CZ, K, AK, IPM, CP, G	1	+	+	+	+
2	7	O_26_:H_11_	Market raw milk	T, OX, E, NA, SXT, AM, CF, CL, K, AK, G	0.786	+	+	+	+
3	8	O_26_:H_11_	Market raw milk	T, OX, E, NA, SXT, AM, CF, CZ	0.571	+	+	+	+
4	22	O_26_:H_11_	Kariesh cheese	T, OX, NA, SXT, CL, K, CP	0.5	+	+	+	+
5	23	O_26_:H_11_	Kariesh cheese	T, OX, E, NA, SXT, CZ	0.428	+	+	+	+
6	25	O_26_:H_11_	Kariesh cheese	T, OX, NA, SXT, AM, CF, CL	0.5	+	+	+	+
7	28	O_26_:H_11_	Kariesh cheese	T, OX, E, NA, SXT, AM	0.428	+	+	+	+
8	36	O_26_:H_11_	Kariesh cheese	T, OX, E, NA, AM, CF, K	0.5	+	+	+	+
9	38	O_26_:H_11_	Kariesh cheese	T, OX, E, NA, SXT, CL, CZ	0.5	+	+	+	+
10	39	O_26_:H_11_	Kariesh cheese	T, OX, E, NA, SXT, AM, CF	0.5	+	+	+	+
11	40	O_26_:H_11_	Kariesh cheese	T, OX, NA, SXT, CZ	0.357	+	+	+	+
12	99	O_26_:H_11_	White soft cheese	T, OX, E, AM, CF, CL, K	0.5	+	+	+	+
13	101	O_26_:H_11_	White soft cheese	T, OX, E, NA, SXT, CZ	0.428	+	+	+	+
14	9	O_26_:H_11_	White soft cheese	T, OX, E, NA, SXT, AM, CF	0.5	+	+	+	+
15	155	O_26_:H_11_	Ice cream	T, OX, E, NA, SXT, AM, CF	0.5	+	+	+	+
16	14	O_128_:H_2_	Market raw milk	T, OX, E, NA, SXT, AM, CF, CL	0.571	+	−	−	−
17	44	O_128_:H_2_	Kariesh cheese	T, OX, E, NA, AM, CF	0.428	+	−	−	−
18	47	O1_28_:H_2_	Kariesh cheese	T, OX, NA, SXT, AM, CL, CZ, K	0.571	+	−	−	−
19	104	O_128_:H_2_	White soft cheese	T, OX, E, AM, CF, CL	0.428	+	−	−	−
20	161	O_128_:H_2_	Ice cream	T, OX, E, NA, SXT, AM, CF	0.5	+	−	−	−
21	162	O_128_:H_2_	Ice cream	T, OX, E, AM, CL, CZ	0.428	+	−	−	−
22	169	O_128_:H_2_	Ice cream	T, OX, E, NA, SXT, K	0.428	+	−	−	−
23	172	O_128:_H_2_	Ice cream	T, OX, E, NA, AM, CF, CL	0.5	+	−	−	−
24	21	O_111_:H_2_	Market raw milk	T, OX, E, AM, CF, CL, CZ	0.5	+	+	+	+
25	32	O_111_:H_2_	Market raw milk	T, OX, E, NA, SXT, AM, CF, CZ	0.571	+	+	+	+
26	48	O_111_:H_2_	Kariesh cheese	T, OX, E, NA, AM, CF, CZ	0.5	+	+	+	+
27	49	O_111_:H_2_	Kariesh cheese	T, OX, E, NA, SXT, AM, CF	0.5	+	+	+	+
28	116	O_111_:H_2_	White soft cheese	T, OX, E, NA, AM, AK	0.428	+	+	+	+
29	119	O_111_:H_2_	White soft cheese	T, OX, E, NA, SXT, AM, CF	0.5	+	+	+	+
30	121	O_55:_H_7_	White soft cheese	T, OX, E, NA, AM, CF	0.428	+	+	−	−
31	123	O_55_:H_7_	White soft cheese	T, OX, E, NA, SXT, AM	0.428	+	+	−	−
32	33	O_55_:H_7_	Market raw milk	T, OX, E, AM, CF, CZ	0.428	+	+	−	−
33	174	O_55_:H_7_	Ice cream	T, OX, E, NA, SXT, AM	0.428	+	+	−	−
34	175	O_55_:H_7_	Ice cream	T, OX, E, NA, AM, CF, CZ	0.5	+	+	−	−
35	183	O_55_:H_7_	Ice cream	T, OX, E, AM, CF, CL, K	0.5	+	+	−	−
36	41	O_119_:H_6_	Market raw milk	T, OX, E, NA, AM, CF	0.428	+	+	−	−
37	42	O_119_:H_6_	Market raw milk	T, OX, E, NA, SXT, AM, CF	0.5	+	+	−	−
38	43	O_119_:H_6_	Market raw milk	T, OX, E, NA, SX, AM, CL	0.5	+	+	−	−
39	16	O_119:_H_6_	Kariesh cheese	T, OX, E, SXT, AM, CF	0.428	+	+	−	−
40	17	O_119:_H_6_	Kariesh cheese	T, OX, E, NA, AM, CF, CL	0.5	+	+	−	−
41	18	O_119_:H_6_	Kariesh cheese	T, OX, E, NA, SXT, AM, CF	0.5	+	+	−	−
42	19	O_119_:H_6_	Kariesh cheese	T, OX, E, NA, AM, CL	0.428	+	+	−	−
43	143	O_119_:H_6_	White soft cheese	T, OX, E, NA, SXT, AM, CZ	0.5	+	+	−	−
44	184	O_119_:H_6_	Ice cream	T, OX, NA, SXT, AM, CF	0.428	+	+	−	−
45	186	O_119_:H_6_	Ice cream	T, OX, E, NA, SXT, AM, CL	0.5	+	+	−	−
46	189	O_119_:H_6_	Ice cream	T, OX, E, NA, SXT, AM, CF	0.5	+	+	−	−
47	190	O_119_:H_6_	Ice cream	T, OX, E, NA, SX, AM, CZ	0.5	+	+	−	−
48	198	O_119_:H_6_	Ice cream	T, OX, E, NA, SXT, CL	0.428	+	+	−	−
49	51	O_159_	Market raw milk	T, OX, E, NA, CF, CZ	0.428	+	−	−	−
50	53	O_159_	Market raw milk	T, E, NA, SXT	0.286	+	−	−	−
51	60	O_159_	Market raw milk	T, OX, E, SXT, NA	0.357	+	−	−	−
52	72	O_159_	Kariesh cheese	T, OX, E, SXT	0.286	+	−	−	−
53	74	O_159_	Kariesh cheese	T, OX, E, NA, SXT, AK	0.428	+	−	−	−
54	78	O_159_	Kariesh cheese	T, OX, E, SXT, CZ	0.357	+	−	−	−
55	81	O_121_:H_7_	Kariesh cheese	T, OX, E, SXT	0.286	+	−	−	+
56	83	O_121_:H_7_	Kariesh cheese	T, OX, E, SXT, K	0.357	+	−	−	+
57	88	O_121_:H_7_	Kariesh cheese	T, OX, E, SXT	0.286	+	−	−	+
58	89	O_17_:H_18_	Kariesh cheese	T, OX, E, CZ	0.286	−	+	−	−
59	201	O_17_:H_18_	Ice cream	T, OX, E, SXT	0.286	−	+	−	−
60	204	O_17_:H_18_	Ice cream	T, OX, E, SXT	0.286	−	+	−	−
61	208	O_17_:H_18_	Ice cream	T, OX, E, K	0.286	−	+	−	−
62	151	O_146_:H_21_	White soft cheese	T, OX, E, SXT, NA	0.357	+	+	−	−
63	91	O_114:_H_4_	Kariesh cheese	T, OX, E, SXT, CL	0.357	−	+	−	−
64	96	O_114_:H_4_	Kariesh cheese	T, OX, E	0.214	−	+	−	−

T: Tetracycline; OX: Oxacillin; E: Erythromycin; NA: Nalidixic acid; SXT: Sulphamethoxazol; AM: Ampicillin; CF: Cefotaxime; CL: Clindamycin; CZ: Cefazolin; K: Kanamycin; AK: Amikacin; IMP: Imipenem; CP: Ciprofloxacin; G: Gentamicin.
